# A Tale of Two Cohorts: Comparing HPV Vaccination Barriers and Facilitators Among HIV-Negative and HIV-Positive Women in Tennessee

**DOI:** 10.3390/ijerph23080977

**Published:** 2026-07-28

**Authors:** Alina Cernasev, Karissa Cliff, Emily Nagel, Hunter Smith, Alex Johnson, Tracy M. Hagemann

**Affiliations:** 1 Department of Clinical Pharmacy and Translational Sciences, College of Pharmacy, University of Tennessee Health Science, Nashville, TN 37211, USA; kcliff@uthsc.edu (K.C.); enagel@uthsc.edu (E.N.); hsmit142@uthsc.edu (H.S.); thageman@uthsc.edu (T.M.H.); 2 Ambulatory Pharmacy, University of Tennessee Medical Center, Knoxville, TN 37920, USA; acjohnson1@utmck.edu

**Keywords:** women living with HIV, HIV-negative women, HPV, HPV vaccine, stories, US, qualitative

## Abstract

**Highlights:**

**Public health relevance—How does this work relate to a public health issue?**

- Physicians play an essential role in the adoption of HPV vaccine adoption for minority women living with HIV (WLHIV) and minority women who are HIV-negative in Tennessee.
- Women living with HIV (WLHIV) are in special need of the HPV vaccine given their likelihood to contract more virulent strains of HPV given their immune compromised status as well as faster progression to malignant cell development.

**Public health significance—Why is this work of significance to public health?**

- This exploration of HPV vaccination revealed that both minority women living with HIV (WLHIV) and minority women who are HIV-negative in Tennessee are largely unaware of the HPV vaccine.
- There is a pronounced lack of understanding about HPV as a disease and limited awareness of the HPV vaccine itself among both cohorts.

**Public health implications—What are the key implications or messages for practitioners, policy makers and/or researchers in public health?**

- Because women living with HIV (WLHIV) experience cervical cancer at 6 times the rate of HIV-negative women, additional HPV vaccine interventions and education are needed for this group.
- Healthcare providers, including physicians, nurses, and pharmacists are in a pivotal position to advocate for and recommend the HPV vaccine to eligible individuals aged 18–45.

**Abstract:**

Background: Human papillomavirus (HPV) continues to cause an estimated 4000 preventable cancer deaths each year in the United States. Given the prevalence of HPV-related disease and suboptimal vaccination rates, this study explores perceptions of HPV vaccination among HIV-negative females and females living with HIV in Tennessee. Methods: This prospective, observational qualitative study consisted of narrative interviews which were conducted with HIV-negative and HIV-positive participants, focusing on their perceptions of HPV and its vaccine. Interviews were conducted by phone, and the verbatim transcripts were inductively coded by three researchers. The inductive codes were then grouped into categories based on similarities which facilitated the emergence of themes. Results: A total of 40 participants were interviewed between July 2024 and December 2024. Narrative analysis revealed three themes: (1) *“I had never heard of it until I saw the flier.”* Gaps in Understanding of HPV and Its Health Implications, (2) Navigating Adolescent Health Decisions and Emotional Influence, and (3) Barriers to Uptake—Skepticism, Trust, and the Need for Tailored Information. Two subthemes emerged: Cohort-Specific Skepticism, and Parental Influence and Communication Gaps. Conclusions: This study revealed significant gaps in HPV knowledge and vaccination uptake among HIV-negative women and women living with HIV in Tennessee. Provider communication was key to vaccine decisions, but clear recommendations were often missed in clinical encounters.

## 1. Introduction

Few infections are as widespread, or as deadly, as human papillomavirus (HPV), which continues to cause an estimated 4000 preventable cancer deaths each year in the United States (US) [1]. More than 85% of people will acquire HPV during their lifetime, and by age 45, over 80% of Americans have already been infected [1,2]. Although most infections are asymptomatic and self-limiting, persistent HPV can lead to anogenital warts, precancerous lesions, and cancers, including cervical, vaginal, vulvar, anal, and oropharyngeal malignancies [1]. Each year in the US, an estimated 36,500 cancer cases are attributable to HPV, with cervical cancer standing out as the most recognized; it is also the only HPV-associated cancer with a recommended screening test for early detection [3]. Approximately 99.7% of cervical cancers are linked to persistent HPV infection, making this a vaccine-preventable disease with a well-defined pathogenesis [4]. In 2022, the National Cancer Institute (NCI) estimated approximately 297,908 US females were living with a cervical cancer diagnosis, with an incidence of 7.7 per 100,000 females annually [5]. Despite available prevention, HPV continues to cause nearly 11,000 cases of cervical cancer, 4000 cervical cancer-related deaths, and an estimated 196,000 cervical precancerous abnormalities each year [3]. The five-year relative survival rate for cervical cancer is 68.0% but increases to 91.4% when disease is detected early via screening tests [5].

HPV vaccination offers a powerful opportunity to change this trajectory, providing highly effective, long-lasting protection against cancers caused by HPV and preventing over 90% of such cancers from developing [3]. The human papillomavirus 9-valent recombinant vaccine (GARDASIL^®^9), approved in 2014, is the only HPV vaccine licensed by the US Food and Drug Administration (FDA) and protects against nine HPV types, including those most linked to cancer [3]. Safety and efficacy have been demonstrated in large clinical trials involving more than 15,000 females and males, extensive post-licensure monitoring, and more than 100 million doses administered nationally since approval [3]. Population-level effects have been substantial. Within four years of introduction, vaccine-type infections dropped by 56% among females aged 14–19, and in twelve years declined by 88% in this group and 81% in females aged 20–24 [3]. Cervical precancer rates declined by 50% in 18–20-year-olds and 36% in 21–24-year-olds among screened females in 2014–2015 compared to 2008–2009, and the proportion of cervical lesions attributable to HPV types covered by the vaccine has fallen by 40% in vaccinated women [3]. Vaccine-induced protection persists for more than ten years without evidence of waning immunity [3]. In people living with HIV (PLWH), HPV vaccines are safe, well tolerated, and generate protective immune responses, supporting their routine use despite somewhat lower antibody levels compared with HIV-negative individuals [6]. Importantly, vaccination before puberty has been shown to elicit the strongest antibody responses and is expected to provide durable protection [3].

Despite its effectiveness, HPV vaccination coverage remains suboptimal. In 2022, only 57.7% of US females aged 19–26 years had received at least one dose, with lower coverage among older age groups [7]. In Tennessee, 77.1% of female adolescents aged 13–17 years had initiated vaccination and 57.4% were up to date, both below national averages [8]. Rural counties report the lowest coverage, with obstacles including access to care, reliable transportation, and healthcare delivery infrastructure [9,10]. In 2020, HPV was responsible for 1089 cancers in the state of Tennessee [11]. While many studies in the literature have examined intervention efforts aimed at pediatric and parent populations, little research has explored the factors affecting HPV vaccine uptake among adults living in Tennessee [11,12,13]. Tennessee represents an important population for vaccination initiatives due to its lower adolescent vaccination rates and higher incidence of HPV-related cancers. These disparities highlight the need for targeted interventions that address the unique barriers faced by adults in this region. Sociodemographic factors, healthcare access, and public awareness may all influence vaccination rates among this population. Understanding these determinants can inform more effective public health strategies and recommendations, such as those from ACIP, that enable individuals to make informed health choices. Given that HPV vaccine coverage in Tennessee remains below the national average, addressing these gaps is essential to improving public health outcomes in the state [9].

HIV-related immunosuppression increases HPV acquisition, persistence, and progression, and is associated with a shift to cervical cancer at younger ages as well as substantially elevated incidence compared with the general population [14]. Coinfection with multiple high-risk HPV types is frequent in PLWH; although HPV16 remains dominant in invasive cervical cancer, HPV18 and HPV45 are relatively over-represented in high-grade disease and cancer in PLWH, informing risk-stratified management [15]. Notably, HPV16 positivity rises steadily with increasing disease severity, from ~13% in normal cytology to nearly 47% in invasive cervical cancer (ICC), confirming it as the most carcinogenic type in this population [15]. HPV18 and HPV45 also show substantial contribution compared with normal findings (ICC: normal ratios 2.47 and 2.55, respectively). Their underrepresentation in cervical intraepithelial neoplasia grade 3 (CIN3) suggests a tendency to cause glandular lesions that may be harder to detect by cytology [15]. Despite a higher burden of multiple HPV infections among PLWH, risk stratification offered by partial HPV genotyping, particularly for HPV16/18 and in some settings HPV45, remains relevant for optimizing screening and management strategies in this population [15]. Antiretroviral therapy (ART) improves CD4 counts and suppresses HIV replication and may reduce high-risk HPV persistence, yet absolute risks for high-grade disease and cancer remain higher in PLWH than in HIV-negative populations [6,14]. Furthermore, the southern US accounts for nearly half of all new HIV infections, and Tennessee faces persistent hurdles with delayed diagnosis and retention in care [16,17].

These intersecting conditions underscore the urgency of enhanced prevention. Current guidance emphasizes intensifying secondary prevention in PLWH with earlier and more frequent cervical screening, partial HPV genotyping to identify highest-risk infections, and consideration of routine anal and vulvar inspection in clinical care [14,15,18]. To support access and uptake, national and local programs, such as the Centers for Disease Control and Prevention (CDC)’s National Breast and Cervical Cancer Early Detection Program and the Nashville and Davidson County Breast and Cervical Screening Program, provide free or low-cost screening and navigation services [19,20]. Nevertheless, current vaccination and screening uptake falls short of what is needed to eliminate HPV-related cancers as a public health threat. Given the prevalence of HPV-related disease, suboptimal vaccination rates, and the compounded risks faced by both HIV-negative females and females living with HIV, this study aims to explore perceptions of HPV vaccination among these two populations in Tennessee.

## 2. Materials and Methods

### 2.1. Study Design

This study employed a prospective, observational, and qualitative approach focusing on women living in Tennessee. This study specifically chose narrative interviews due to their numerous advantages. Narrative interviews allow participants to share their experiences in their own words, which provides a rich and in-depth understanding of their perspectives [21]. Contrary to structured interviews that often limit responses to predefined categories, narrative interviews elicit open-ended storytelling. This approach aligns with the study’s aim to explore the intrinsic and extrinsic motivators for HPV vaccination among women living with HIV and those who are HIV-negative in Tennessee. It emphasizes the meanings and interpretations participants attach to their experiences rather than focusing solely on factual details.

The choice of narrative interviews also enabled the research team to capture the temporal and contextual dimensions of participants’ experiences [22]. Through storytelling, participants naturally organize their accounts around significant moments, in this case receiving or not receiving the HPV vaccine, turning points, and relationships, which reveal how their experiences evolve over time. This structure provides valuable insights into processes of change and decision-making, which are elements often not captured in other forms of data collection such as quantitative studies.

Furthermore, narrative interviews foster a more collaborative and reflexive relationship between researchers and participants [22]. By allowing participants to guide the flow and focus of their stories, the interviewer assumes the role of an active listener rather than a rigid questioner. This change in dynamics builds trust and openness, facilitating a deeper reflection and an explanation of the decision-making process [23]. The flexibility of this narrative approach is well-suited for exploring sensitive or complex topics, as participants can choose how to frame and disclose their experiences [23].

### 2.2. Participants and Settings

The participants in this study were carefully selected to ensure their experiences were directly relevant to the research question. After receiving IRB approval from the University of Tennessee Health Science Center, recruitment began through the distribution of flyers in the English language. Flyers were placed in libraries, Hispanic commercial centers, and in lounges and bars. Since the flyers were in English, fluency in English was necessary for participation, and this requirement may have limited participation by excluding non-English speaking women. The study consisted of two arms, starting with the recruitment of HIV-negative women living in Tennessee [24]. Once data was collected from this group, recruitment for HIV-positive women from Tennessee commenced [25]. Despite flyer placement in the same locations for both arms of the study, the racial demographic for the WLHIV arm of the study was composed entirely of African American women, compared to the HIV-negative arm, which also included Asian and Latina women. The majority of individuals living with HIV in Tennessee are disproportionately (*p* < 0.001) Non-Hispanic Black (56.3%); the other groups living with HIV in Tennessee are Non-Hispanic Whites (34.0%), Hispanic (6.7%), and Other/Unknown (3.0%) [12]. Since the number of Hispanic and Asian individuals living with HIV is significantly smaller than the Non-Hispanic Black individuals, this likely contributed to the predominance of Non-Hispanic Black women in the WLHIV arm.

### 2.3. Eligibility Criteria

Arm 1: HIV-negative participants: women residing in Tennessee at the time of study, age 18–45, regardless of their HPV vaccination status; English-speaking, and willing to share their perspective about the HPV vac cine.

Arm 2: HIV-positive participants: women residing in Tennessee at the time of the study, HIV diagnosis and taking antiretroviral treatment, regardless of their HPV vaccination status, 18–45 years old, English-speaking, and willingness to discuss HPV vaccination perspectives after HIV seroconversion.

Exclusion criteria: Minors < 18 years of age, self-identifying males, without access to a reliable mobile phone to be contacted and participate in the study, plan to leave the area for an extended period of time in the next 6 months, and/or non-Tennesseans.

### 2.4. Recruitment

Recruitment was conducted through targeted outreach within relevant minority communities and professional networks. Flyers were placed on community boards in churches, universities, restaurants, markets, libraries, HIV centers, and clinics throughout Tennessee. Participants were invited to take part in the study, and those who expressed interest received detailed information about the study’s purpose, confidentiality procedures, and the voluntary nature of their involvement.

Interested individuals provided a phone number or email address, which the research team used to contact them and screen for eligibility criteria. Once eligibility was confirmed, the research team scheduled a phone interview at a convenient time for the participants. Informed verbal consent was obtained from all participants before data collection began. During the phone interviews, it was ensured that participants felt comfortable and empowered to share their experiences openly, recognizing the deeply personal and reflective nature of narrative interviews. At the end of each complete interview, the participants received a $75 gift card.

### 2.5. Data Collection, Analysis, and Rigor

Data collection for this study was conducted through thematic analysis of narrative interviews aimed at eliciting detailed stories from participants [13,22]. At the beginning of each interview, participants’ demographic information was collected. Each interview commenced with an open-ended prompt that encouraged participants to share their experiences in their own words, allowing their narratives to unfold naturally. More details regarding the interview questions can be found in the previous publications. [24,25]. Follow-up questions were used sparingly to encourage elaboration or clarification without disrupting the flow of the participants’ storytelling and to preserve consistency between interviews [13,22].

In the second arm of the study, the narrative interviews focused on participants’ HIV diagnoses and their views on antiretroviral treatment. Interviews in both arms of the study were conducted until thematic saturation was achieved, typically lasting between 30 and 90 min [13,22]. With the participants’ consent, the interviews were audio-recorded to ensure accuracy.

Once collected, the data was transcribed verbatim by a third party to preserve the participants’ exact words and expressions [13,22]. The data was imported into Dedoose^®^, (Version 9.0.17, Los Angeles, CA, USA) a qualitative software that facilitated the organization of codes, categories, and memos. The thematic analysis employed a narrative-focused approach focusing on the participants’ stories, the manner of their narration, and the relevance to intrinsic and extrinsic motivators for receiving the HPV vaccine. Transcripts were read multiple times to gain familiarity and were inductively coded by two researchers [13,22]. The research team convened to review the codes, categories, key themes, and narrative patterns. Each participant’s story was analyzed holistically to understand how they made sense of their experiences, constructed their identities within their narratives, and how broader social or contextual factors influenced their accounts. The emerging themes were refined and compared across participants from both arms of the study to capture both individual and shared experiences [13].

**Figure 1:**
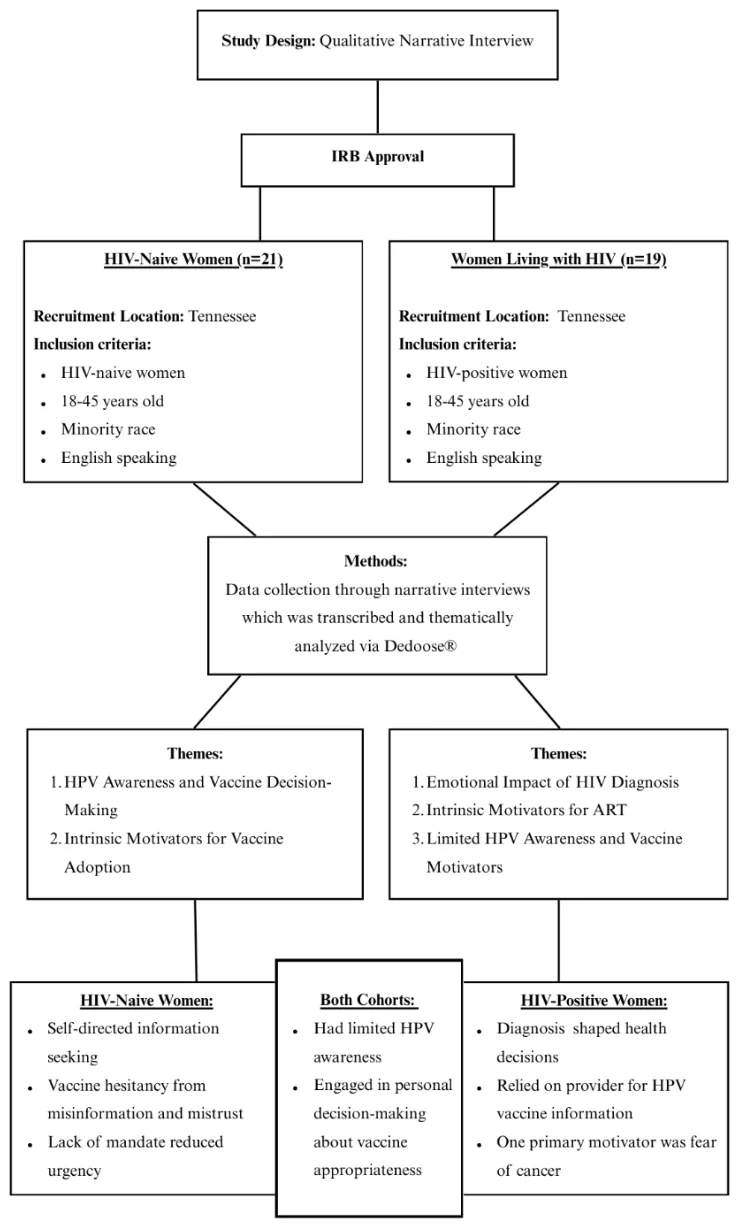
Methodology and results.

To ensure rigor and trustworthiness, several strategies were employed following Lincoln and Guba’s framework [26]. Credibility was achieved through prolonged engagement with the data, memo writing, and coder checks, where the research team discussed the findings [26]. Dependability and confirmability were supported by maintaining an audit trail documenting all codes, categories, and analytical steps. Rich, detailed descriptions of participants’ contexts and experiences were provided to enhance transferability, allowing readers to assess the relevance of the findings in other contexts [26]. A detailed presentation of the methodology can be found in Figure 1.

## 3. Results

A total of 40 participants were interviewed in this study, including 21 HIV-negative women and 19 women living with HIV (WLHIV). The HIV-negative cohort had a mean age of 28 years and included African American (*n* = 11), Latina (*n* = 7), and Asian (*n* = 3) participants, while the HIV-positive cohort had a mean age of 39 years and all participants identified as African American, reflecting the predominance of this group LHIV in Tennessee. The collected data led to the emergence of three themes.

**Theme 1.**  *“I had never heard of it until I saw the flier.”Gaps in Understanding of HPV and Its Health Implications*.

A central theme that emerged from the narrative interviews was a pronounced lack of understanding surrounding HPV as a disease. Many participants, within both the HIV-negative cohort and some of the HIV-positive cohort, remained unaware of the connection between HPV and cervical cancer. This lack of awareness hindered participants from comprehending the essence of HPV, its modes of transmission, or the importance of vaccination. During the narrative interviews, the interviewer emphasized that there was no right or wrong answer and that participants could share whatever they were comfortable with.

The following quotes illuminate the gaps in knowledge, revealing that participants’ main sources of information were advertisements, social media, or word of mouth. Several participants expressed that they were encountering HPV for the first time:

*“I’m not sure because this is my first time hearing of it.”*

(WLHIV4)

*“The only thing I know is what I see on commercials and ads… and also on social media, like Instagram.”*

(HIVneg12)

*“The only time I’ve heard about it was from your flier.”*

(HIVneg20)

Beyond complete unfamiliarity, some participants held various misconceptions about HPV, describing it as a skin condition, believing it spreads through casual touch, or expressing uncertainty about who it affects:

*“I think it’s like maybe a virus that has something to do with your skin… maybe a type of cancer.”*

(HIVneg9)

*“I know it’s an STI… as easy as touching a person who has it.”*

(HIVneg1)

*“I believe it’s women. I guess I don’t know for sure.”*

(HIVneg3)

This unfamiliarity was reflected not only in their responses but also in their surprise at encountering HPV information for the first time:

*“Okay. I think a virus, dangerous. I am scared [of] this.”*

(HIVneg10)

*“I feel like it might be a dangerous virus. I’m not really sure.”*

(HIVneg11)

Some participants confused HPV with HIV, revealing the extent of misunderstanding about these distinct viruses. When asked about HPV, these participants offered descriptions of HIV as an incurable disease linked to sharing needles and unprotected sex, illustrating the depth of confusion between the two viruses:

*“It kind of almost sounds like HIV. Oh, God. I don’t even want to say that disease, but that’s the incurable, that you don’t want… I think that’s caused by like sharing needles and unprotected sex.”*

(HIVneg15)

*“Right away I want to say it’s HIV. That’s the first thing that comes to my mind. Like a sexual disease.”*

(HIVneg6)

*“Well, I’m assuming that HIV, well, HPV sounds like a type of STD, so I’m assuming like sexually transmitted.”*

(HIVneg9)

Another participant described her limited knowledge about HPV, expressing uncertainty about what it actually is and associating it only with vague effects on reproductive organs:

*“Well, when I first heard of it, I wasn’t exactly sure what it was. I thought that it was something that can somehow affect your reproductive organs, but that’s pretty much the only knowledge I’ve had of HPV.”*

(HIVneg5)

This widespread lack of understanding about HPV has important public health implications. Without knowledge of what HPV is, how it spreads, or its connection to cervical cancer, individuals are ill-equipped to make informed decisions about vaccination. The reliance on advertisements and social media, rather than healthcare providers or comprehensive health education, further compromises public health efforts to increase HPV vaccination rates and prevent cervical cancer.

**Theme 2.**  *Navigating Adolescent Health Decisions and Emotional Influence*.

A second theme emerged that illuminated the factors influencing HPV vaccination decisions among the HIV-negative cohort. Since some of the participants were vaccinated as adolescents the discussion focused on recalling those health decisions, particularly around emotional pressure and evolving understanding.

Participants highlighted a pervasive gap in sexual health education. Young people recounted experiences where the rationale behind vaccines was not fully explained, leaving them uncertain and reliant on partial information from peers or healthcare providers:

*“I remember them saying that, if I was to be sexually active… this vaccine will completely stop your chances or help with your chances of like not having that. And I think that was a scare tactic for me because this was all new information and it made me respond emotionally rather than informed.”*

(HIVneg4)

*“Well, when I was younger, I actually told my parents that I was thinking about having sex, and so when I did that, it prompted us to calling to meet a gynecologist… she was informing me of what birth control would prevent and then was also like, you know, going into STDs… saying that I guess like the medical world was getting a little bit more familiar with HPV and that I should probably get a vaccination before I started having sex.”*

(HIVneg8)

These quotes express a recurring hope for better education that demystifies vaccines and empowers adolescents to make decisions based on knowledge rather than fear:

*“… it was just weird that I had to go and get [the vaccine] because I’m not really educated on like why you need vaccines. Maybe when you were a newborn, yes. But like in middle school, I don’t understand why… I do believe like, oh, it can help you like… maybe have a stronger immune system. Maybe. Like it maybe can help you not spread any type of diseases.”*

(HIVneg9)

*“The first thing that comes to my head is, if someone says HPV vaccine, the first thing I would say is go get it, and then the education that comes with it. But, unfortunately… not everyone’s educated on anything half the time.”*

(HIVneg16)

These accounts illustrate a tension between emotional responses and informed decision-making in adolescent health. Participants described feeling pressured, scared, or confused rather than empowered and educated. This reliance on fear-based messaging and incomplete information, rather than comprehensive health education, suggests missed opportunities to engage young people as active participants in their own healthcare decisions. The findings underscore the need for age-appropriate, comprehensive HPV education that reaches adolescents before vaccination decisions arise. Beyond these educational gaps, additional barriers further complicated vaccination decisions for both cohorts.

**Theme 3.**  *Barriers to Uptake—Skepticism, Trust, and the Need for Tailored Information*.

A dominant theme across both cohorts centered on vaccine hesitancy, rooted in limited prior vaccination experiences, distrust in medical systems, and concerns about safety, particularly among women living with HIV.

### 3.1. Limited Vaccination Experience and Fear of Harm

Some WLHIV participants reported receiving only childhood immunizations and having little to no engagement with adult vaccinations such as COVID-19 or flu shots. This lack of familiarity contributed to uncertainty about vaccine purpose and safety:

*“The only vaccinations I’ve ever had was the ones we do as little kids, infants.”*

(WLHIV13)

*“The only vaccines I remember receiving are the ones that you get as a little—as a kid, like the polio shot, the shots for the measles… Any other vaccines, I have not… I haven’t gotten any of the COVID shot vaccines at all.”*

(WLHIV10)

Fear of harm emerged as a major driver of hesitancy across both cohorts. Participants expressed concerns about what vaccines contain, how they affect the body, and their potential side effects. Many articulated a deep mistrust towards governmental and pharmaceutical intentions, heightened by concerns about the unprecedented speed of COVID-19 vaccine development and the influence of negative media coverage. These factors converged to amplify doubts regarding the reliability and transparency of public health messaging. One participant’s analogy to “chemical warfare” poignantly illustrates the intensity of this skepticism:

*“I have a problem with the fact that they actually take the virus itself and inject it… and it makes sense, to build an immune system against it, but also just like I think of it as chemical warfare.”*

(WLHIV13)

*“How does the things that we’re putting in our body, how does it affect the body? Like short term, long term… I’ve heard horror stories.”*

(HIVneg1)

Despite this skepticism and vaccine hesitancy, participants demonstrated a desire for reliable, personalized information, especially from trusted healthcare providers. Some WLHIV participants specifically emphasized wanting guidance from their physicians, with whom they have an established rapport, to understand risks and safety in the context of HIV. Access to clear, credible information was seen as essential to making an informed choice.

### 3.2. Cohort-Specific Vaccine Skepticism

Although skepticism in vaccines was persistent in both cohorts, notable differences emerged. The HIV-positive cohort expressed heightened concerns influenced by their chronic illness experiences, frequent interactions with healthcare providers, and fears about potential side effects or drug interactions with their existing medications. However, this skepticism was often balanced by a genuine desire to safeguard their own health and serve as role models for others in their community.

WLHIV expressed concerns about how the vaccine would affect their compromised immune systems, while simultaneously grappling with trust issues regarding the information they receive:

*“Like what the side effects are with someone with my health condition, how would it play a factor. I already have a compromised immune system, like am I protected? What are my risk factors, the percentage of the risk factors?… And I know I’m skeptical, too, because I believe in our government, but I don’t… are they really telling me the truth? Am I taking something that’s going to cause additional health issues?”*

(WLHIV10)

Beyond individual health concerns, some WLHIV participants described how their perspective on vaccination was evolving, motivated by the potential to serve as examples for others facing similar health challenges:

*“The benefits I think would be I wouldn’t have to worry about catching the HPV virus and possibly developing cancer. I’m living a healthier, longer life, being HIV positive on top of that… I could be a role model for other individuals to go ahead and get the vaccine… I’m trying to change my perspective on it for the simple fact that, if other individuals can see someone of color that’s HIV positive that is doing it, then they will be more inclined to do it as well, I think.”*

(WLHIV13)

In contrast, the HIV-negative cohort displayed skepticism rooted more in general vaccine hesitancy and misinformation rather than personal chronic health experiences. The skepticism in this cohort stemmed from fear of side effects, deep-seated distrust in vaccine safety, and anxiety about needles or the perceived risks of vaccination. These concerns were often magnified by skepticism following the COVID-19 pandemic:

*“I was scared… maybe I thought it was going to have side effects.”*

(HIVneg15)

*“One of my biggest fears was Guillain-Barré syndrome.”*

(HIVneg17)

*“I don’t know what is in it. I don’t like getting vaccines I don’t know much about.”*

(HIVneg20)

*“After my aunt got the COVID vaccine, she passed away… it just turned me away from vaccines.”*

(HIVneg12)

Another difference between the cohorts was health literacy. In the HIV-negative cohort, some women did not comprehend the relationship between HPV and cervical cancer. Women with less education or poor access to health information may not fully understand what HPV is, how it spreads, or the importance of receiving the vaccine before becoming sexually active:

*“I don’t really think of the vaccine much because I didn’t get one as a kid. And so I guess like the word that comes to mind would be like unnecessary.”*

(HIVneg1)

*“To be honest, I don’t know much about the vaccine.”*

(HIVneg12)

Within the WLHIV cohort, understanding the link between HPV and cancer was more straightforward, as participants regularly consulted with their physicians. Fear of side effects contributed to hesitancy in both cohorts. Even minor adverse effects, including fever or soreness at the injection site, can be amplified by rumor or misunderstanding, leading women to overestimate risks compared to benefits. These distinctions between cohorts illustrate the need for tailored communication strategies to address each group’s unique concerns.

### 3.3. Parental Influence and Communication Gaps

Parental influence, especially from mothers, played a central role in vaccination decisions for both cohorts, often accompanied by limited communication and mixed attitudes toward the vaccine’s necessity. HPV vaccination decisions were influenced by a combination of parental perspectives, personal comfort levels, and varying degrees of health literacy. While some individuals deferred to family decisions due to age or uncertainty, others expressed a willingness to follow recommended vaccination practices despite limited knowledge.

Some participants felt excluded from the decision-making process entirely, with parents also left uninformed by healthcare providers:

*“Yeah, I think my mom was kind of left in the dark with that too… I remember getting into the car and I told her I got that shot and I need to come back, and she was like, why did you get that shot? And she was asking for information, so I gave her the paper. And she seemed annoyed by it… like, well, it’s too late now… I feel like the ideal situation would have been my mom would have been there and probably would have helped me in that decision… I knew a little bit about sexual health and stuff like that, but I didn’t really know a lot about it… I would have wanted to be informed and then go in with my mom and know the risks of… what will happen after taking the shot.”*

(PHIVneg3)

Some individuals deferred to family decisions despite their own ambivalence or lack of understanding:

*“My mom was totally against it, I’ll tell you that. But, at the end, I think it became a requirement, or I’m not sure, but she did end up taking me to go get it… I’m going to be honest, I really didn’t care if I got it or not. My mom was the one that was mainly like against it… at some point, she just decided that, yeah, that it would be best for me to get it. I guess because I was at an age where like, you know, you become sexually active. So maybe that was her motivation behind it.”*

(HIVneg4)

Within the WLHIV cohort, participants emphasized the importance of education and advocacy, urging parents to actively encourage their children to receive the vaccine:

*“They should teach it more… a lot of parents… are not familiar with the HPV, so they need to like educate the kids or the parents more.”*

(WLHIV 19)

*“… that was one of the main things concerning where is with my daughter having kids, to make sure that she get the HPV shot.”*

(WLHIV11)

One HIV-positive participant described feeling traumatized by fear-based messaging during her vaccination encounter, demonstrating the emotional impact that communication styles can have on patients:

*“… it was also kind of traumatizing because I felt like I was scared into getting it… They were telling me the worst-case scenarios… they were just like, you should definitely get it just in case, it’s covered by insurance, blah, blah, blah. And so I ended up getting it and then having to come back for another round. But it hurt my arm so bad that I never came back.”*

(WLHIV3)

These experiences highlight opportunities to strengthen communication and shared decision-making during clinical encounters. Ensuring that adolescents and their parents receive clear, timely information about the HPV vaccine and potential side effects may help families feel more prepared and supported. By improving transparency and inviting caregivers into the process when appropriate, clinics can foster trust and reduce confusion for patients navigating important health decisions.

## 4. Discussion

This qualitative study examined HPV vaccination perceptions among WHLIV and HIV-negative women in Tennessee. Our findings reveal interconnected barriers and facilitators that shape vaccination decisions, including knowledge gaps, provider communication quality, family dynamics, and structural access issues. Collectively, these influences determine whether HPV vaccination is initiated, delayed, or completed. These findings align with existing evidence that HPV vaccination decision-making is shaped by multilevel influences spanning individual knowledge, interpersonal dynamics, and healthcare system interactions [27,28,29].

Vaccine adoption in general involves complacency, convenience, and confidence [30]. Confidence was key for the participants in this HPV-vaccine study, particularly as it related to their own health care professional in terms of actual HPV vaccine adoption [30]. Complacency was evident in that participants did not see direct benefits for the HPV vaccine for what, to them, was an unknown virus with little immediate impact, since harm wasn’t seen until decades later [30]. Convenience played a role for vaccine adoption for the study participants, in that during the traditional HPV vaccination schedule window—starting at age 9 years to age 26 years—the vaccination was not mandatory and for some was an unaffordable expense even if they had awareness.

### 4.1. Knowledge Gaps and Health Literacy

The knowledge deficit among participants was striking. Women perceived the vaccine as “unnecessary” if not received during childhood (HIVneg1), revealing fundamental misunderstanding of both HPV transmission and catch-up vaccination value. Some participants encountered HPV information for the first time through study recruitment materials, while others confused HPV with HIV or described it vaguely as “something that can somehow affect your reproductive organs” (HIVneg5). These misconceptions about vaccine eligibility or confusion between viruses reflect a fragmented understanding of HPV and its vaccine. Prior work has shown that individuals may recognize HPV terminology while lacking the depth of knowledge needed to make informed decisions [28,31]. Studies across diverse populations have documented persistent gaps in understanding HPV transmission, vaccine eligibility, and cancer risk, even among individuals who report general awareness [28,31,32,33].

Among WLHIV, knowledge appeared somewhat higher, likely reflecting more frequent engagement with healthcare providers. However, even within this group, participants did not consistently connect HIV status with increased susceptibility to HPV-related cancers. This suggests that routine care interactions are not fully leveraging the opportunity for risk-specific education. Prior research similarly indicates that having access to care does not guarantee receipt of clear or actionable vaccine information, as gaps in provider communication, incomplete explanations, and limited translation of clinical guidance into patient understanding persist even among individuals engaged regularly in healthcare [27,28,29,34]. This finding underscores the need for more intentional communication strategies that translate clinical risk into meaningful patient understanding.

#### Structural and Socioeconomic Factors

Differences in social determinates of health (SDOH) between the HIV-negative and WLHIV arms showed wide disparities that may have contributed to participants perceptions of vaccination and health care. In both arms, all were urban participants, but differences in the education level in the HIV-negative group, which included an assortment of minority women with educational attainment ranging from high school graduates to doctoral degree-holders, differed from the WLHIV arm in whom the highest attained level of education was a high school diploma. The women with highest educational levels tended to be of Asian descent with all holding at least a college degree and they were a small subset of the HIV-negative arm (*n* = 3). As a whole, the WLHIV arm had lower socioeconomic status which correlated with their education levels [35].

There was a somewhat paradoxical ability to access healthcare between the two groups. Once women received an HIV-diagnosis they were able to receive good quality, consistent care. Some women in the HIV-negative group did not have consistent care but the majority did. In the HIV-negative group, some participants, especially Latina women, did not have consistent access to care and vaccinations, especially once they came to the United States. Access to care did seem to positively influence the women’s trust in healthcare providers and their recommendations which led to eventual HPV vaccine adoption.

### 4.2. Provider Communication and Missed Opportunities

Provider communication emerged as one of the most influential and inconsistent factors shaping vaccination decision-making. The majority of healthcare providers that the respondents mentioned were nurse practitioners, pediatricians, and general practitioners who all would have been adequately trained regarding the HPV vaccine and its importance and benefits. While the prior literature has established provider recommendation as a key driver of HPV vaccination uptake, these findings extend that understanding by illustrating how the quality and framing of communication influence not only initiation but also completion [29,34,36]. Fear-based messaging appeared to prompt initial uptake but discouraged completion, suggesting that how recommendations are delivered may influence sustained engagement with vaccination.

At the same time, many participants described never receiving a recommendation despite healthcare encounters during eligible periods. Missed opportunities for HPV vaccination in clinical settings have been consistently documented, including cases where patients interact with healthcare providers but are not offered or encouraged to receive the vaccine [34,36]. For individuals in the catch-up age range, these gaps may be compounded by assumptions about prior exposure or reduced benefit.

Message framing also played a role. Evidence indicates that framing HPV vaccination around cancer prevention, rather than focusing on transmission, may improve receptivity and uptake [36,37]. In this study, participants’ experiences suggest that clarity, tone, and delivery are critical components of effective communication.

### 4.3. Family Influence and Communication Dynamics

Family dynamics, notably maternal involvement, were another important influence on vaccination decisions. Participant accounts demonstrated how gaps in provider communication extended beyond the individual to affect family-level decision-making. When parents were not adequately informed, they were less able to support or guide vaccination decisions, sometimes resulting in frustration or disengagement.

Research has similarly shown that parents play a central role in adolescent vaccination decisions but often feel underinformed or unsupported when making these decisions [34,36,38,39]. Broader work examining family and cultural contexts highlights how uneven access to information within families can shape health behaviors and decision-making processes [27,33,40]. Strengthening provider communication may therefore have downstream effects that improve family engagement and support.

### 4.4. Vaccine Hesitancy

Vaccine hesitancy emerged as a cross-cutting theme, shaped in part by broader sociocultural influences, including experiences during the COVID-19 pandemic. Participants’ concerns about safety, trust, and long-term effects were not limited to HPV vaccination but reflected more generalized skepticism toward vaccines, which had broadened due to the COVID-19 pandemic with mandated vaccinations. Some participants had experienced loss of loved ones during the pandemic and linked the vaccinations with their deaths rather than the emergence of more virulent strains. Additionally, government-mandated vaccines remained a source of lingering mistrust for some African American participants in Tennessee. These generational fears appeared to have been passed down through anecdotal discussions with older members of the participants’ extended families.

Similar patterns have been observed in studies examining HPV vaccine attitudes among adult women, where safety concerns, mistrust, and perceived risk influence decision-making [41,42,43,44,45]. Among WLHIV, hesitancy was further shaped by concerns about immune status and uncertainty regarding vaccine safety in the context of chronic illness. Some participants described transforming this hesitancy into motivation to receive the vaccine and serve as role models for their communities, suggesting that social identity and peer influence may also function as facilitators. Prior research has identified the role of social norms, perceived responsibility, and community context in shaping vaccination behaviors, particularly among populations disproportionately affected by HPV-related disease [27,46,47].

### 4.5. Implications for Practice and Intervention

Taken together, these findings suggest that improving HPV vaccination uptake requires coordinated efforts across multiple levels. In clinical settings, providers should deliver clear, consistent recommendations while emphasizing the continued benefit for cancer prevention, catch-up vaccination and avoiding fear-based messaging. Evidence supports the importance of strong provider engagement and effective communication strategies in improving vaccine uptake [41,48,49,50,51]. In terms of cancer prevention, cervical cancer screening is recommended in the US regardless of HPV vaccination with cytology and high-risk HPV testing done in appropriate intervals based on age until ages 65 pending negative screenings prior [52]. Appropriate vaccine stewardship can empower clinicians to encourage routine HPV vaccination as early as 9 years of age, and should continue to be strongly recommended as catch-up vaccinations until 26 years of age; shared medical decision making for HPV vaccination may be conducted up to 45 years of age. Patients living with HIV should receive 3-dose HPV vaccine series regardless of age secondary to immune response attenuation [3,53]. Language emphasizing the importance of vaccination as primary protection sculpt patient friendly conversations focused on education opposed to fear of illness.

Beyond clinical care, community-based and peer-informed approaches may help address persistent knowledge gaps and build trust Multilevel interventions incorporating education, communication, and social support have been proposed as effective strategies for improving HPV vaccination outcomes [29,40,44,45,54]. Identifying peer-advocates for these strategies within both the HIV-negative group and WLHIV group may be an effective vaccine campaign strategy. Trained peer educators appeared to have a positive influence on the few participants who had experienced this type of program. For WLHIV, integrating HPV education into routine HIV care may provide a key opportunity to deliver tailored, risk-specific information [47,55,56,57].

### 4.6. Strengths, Limitations, and Future Research

This manuscript, which employed narrative inquiry among a mixed cohort of WLHIV and HIV-negative women, contributes to the HPV literature by providing insight into how vaccination decisions are made across differing risk profiles. The use of a narrative approach allowed for an in-depth exploration of participants’ lived experiences, capturing perspectives that are often not reflected in quantitative work and highlighting the factors influencing vaccine decision-making within this population.

Recruiting WLHIV and facilitating open discussion of HPV-related experiences presented challenges, and some participants may have been hesitant to fully disclose their perspectives in conversations with researchers. As a result, the narratives presented here may not reflect the full range of experiences among women in Tennessee or among WLHIV more broadly.

Building on these findings, future research could extend this work by including additional purposively selected populations, such as men living with HIV in Tennessee, to further examine how HPV vaccination perspectives may vary across groups. Expanding to mixed-methods designs may also help contextualize these qualitative insights within broader patterns of vaccine uptake and coverage.

The findings also underscore the importance of developing targeted public health strategies for women approaching the upper end of the recommended vaccination age rage, regardless of HIV status. Tailored approaches that address knowledge gaps, communication barriers, and concerns identified in this study may improve engagement with HPV vaccination. Healthcare providers, including physicians, nurses, and pharmacists, remain well-positioned to support these efforts through consistent and clear vaccine recommendations to eligible individuals aged 18–45. Prior research has demonstrated that provider engagement is associated with higher vaccination uptake, including among women in Tennessee [42]. Continued efforts to strengthen provider communication and expand access to accurate information will be important for reducing the burden of HPV-associated cancers in this population.

## 5. Conclusions

This study reveals significant gaps in HPV knowledge and vaccination uptake among both HIV-negative women and WLHIV in Tennessee. Despite the proven safety and efficacy of HPV vaccination in preventing related cancers, participants across both cohorts demonstrated limited understanding of HPV transmission, vaccine eligibility, and the connection between HPV and cervical cancer. Provider communication emerged as a key determinant of vaccine decision-making, yet opportunities for clear, consistent recommendations were frequently missed in clinical encounters. These findings highlight the urgent need for tailored communication strategies that address the unique concerns of each population.

Addressing Tennessee’s persistently low HPV vaccination rates will require coordinated efforts across multiple levels of the healthcare system. Clinicians must deliver strong, evidence-based recommendations while avoiding fear-based messaging that may discourage series completion. For WLHIV, integrating HPV education into routine HIV care offers a critical opportunity to provide risk-specific information and emphasize the heightened cancer risk associated with HIV-HPV coinfection. Community-based interventions, peer support networks, and culturally responsive outreach may help address persistent knowledge gaps and build trust among populations experiencing vaccine hesitancy. By strengthening communication, expanding access to accurate information, and empowering women to make informed decisions, Tennessee can reduce the burden of preventable HPV-associated cancers and move toward eliminating these diseases as a public health threat.

## Data Availability

The original contributions presented in this study are included in the article. Further inquiries can be directed to the corresponding author.
